# Retrospective study on the emotional status of healthcare workers in a COVID-19 field hospital in Oman

**DOI:** 10.3389/fpubh.2024.1339703

**Published:** 2024-05-21

**Authors:** Roopa Koshy McCall, Hamed Al-Sinawi, Nutaila Al-Kharusi, Sulaiman Al Rawahi, Rola Al Balushi, Nabil Al-Lawati, Manfredi Rizzo, Khalid Al-Rasadi, Abdullah Al Maniri

**Affiliations:** ^1^Al Harub Medical Center, Muscat, Oman; ^2^Department of Behavioral Medicine, Sultan Qaboos University Hospital, Muscat, Oman; ^3^PDO Clinic, Corporate Health & Safety, MSEM, Petroleum Development of Oman, Muscat, Oman; ^4^Ministry of Health, Muscat, Oman; ^5^Department of Health Promotion, Mother and Child Care, Internal Medicine and Medical Specialties (Promise), University of Palermo, Palermo, Italy; ^6^Department of Biochemistry, College of Medicine and Health Sciences, Sultan Qaboos University, Muscat, Oman; ^7^Medical Research Centre, Sultan Qaboos University, Muscat, Oman; ^8^Oman Medical Specialty Board, Muscat, Oman

**Keywords:** healthcare worker (HCW), Oman, COVID-19, field hospital, psychological status

## Abstract

**Overview:**

To combat the overwhelming demand for medical services and care during the COVID-19 Pandemic, the Sultanate of Oman launched the COVID-19 Field Hospital in 2020, designed to respond and alleviate the burden on the medical infrastructure. Several studies globally and from the Middle East suggested that frontline healthcare workers (HCW) were at risk of developing markers of psychological distress. It was further understood through research findings that HCW were resilient during times of crisis. However, there is a dearth in studies evaluating the emotional status of frontline HCW posted in the COVID-19 field hospitals in Gulf Countries, including Oman. This study attempts to shed light on the emotional status of HCW that were on the frontlines in the field hospital in the Sultanate of Oman.

**Aim:**

This study aims to quantify and evaluate the emotional status of HCW in the frontline field hospital by screening for symptoms of depression, anxiety, and sleep quality.

**Method:**

The data was collected by a local private mental healthcare facility as part of digital feedback to design and implement supportive strategies. Data was collected between September 2021 and October 2021 from 121 HCW in the COVID-19 Field hospital in Oman via ‘WhatsApp’.

**Results:**

Chi square and binary logistic regression tests were administered to evaluate the data. The participants comprised of 63.6% females and 79.3% were between 30 and 39 years of age. Majority of the participants (65.2%) described themselves as ‘financially unstable’ and possess an average of 7.5 years of work experience. Of the participants 73.6% of the HCW were based solely in the field hospital for 6–9 months at the time of the survey. Majority of the participants denied the presence of emotional distress expressed through depression (92.6%), anxiety (92.6%) and poor quality of sleep (59.5%).

**Conclusion:**

The findings of the present study reflect the HCW ability to cope during challenging situations likely owing to a variety of environmental, social and personal protective factors. The findings of this study can translate into further research on identifying and addressing stressors and targeting the enhancement of protective factors to safeguard the well-being of HCW.

## 1 Introduction

The World Health Organization (WHO) declared the COVID-19 outbreak as a public health emergency and a global pandemic in March 2020. Despite COVID-19 no longer being classified as a ‘Global Health Emergency’ by the WHO since May 2023 (1), literature has emerged highlighting the need for preservation of the well-being of healthcare workers (HCW) during crisis (2). As per the consideration of the WHO, HCWs are a classification of “all people primarily engaged in actions with the primary intent of enhancing health.” This includes all individuals involved in activities aimed at improving health (3). Past literature on pandemics has highlighted the negative emotional status of healthcare workers (HCWs), stemming from increased workload, uncertain hospital procedures and policies, and perceived risk of infection and stigma (4).

Emotional Status’ is regarded as the feelings experienced by an individual or a collective group in relation to a particular situation, which may encompass psychological distress (5). Since the onset of COVID-19, findings have emerged recognizing and highlighting the prevalence of psychological distress among HCW during the pandemic (6–8). Similarly, numerous cross-sectional studies reported high rates of depressive and anxiety symptoms, as well as sleep disturbances and burnout among HCWs (9–12). The shortage of HCWs, coupled with hospitals operating beyond their capacity, has contributed to burnout and exhaustion among HCWs (13, 14). Due to hospitals being overwhelmed by the sheer volume of patients and the consequent impact on the well-being of HCWs, field hospitals have emerged as a recognized and strategic tool used by countries to alleviate the burden on established health facilities and mitigate the spread of the virus in the community (15, 16).

Field hospitals have a long history in military contexts, where they serve as temporary, mobile medical facilities designed to triage, stabilize, resuscitate, and provide acute care to wounded soldiers near battlefields (17). The Oman News Agency (18) reported that on October 10, 2020, the Ministry of Health (MOH) in Oman launched a COVID-19 field hospital at the location of the former Muscat International Airport, which held a capacity of over 300 beds and offered full-time management of mild, moderate, and severe cases of COVID-19. The hospital was equipped with a pharmacy, laboratory, and radiology department with over 40 doctors, 200 nursing staff, and a range of other healthcare professionals such as lab technicians, pharmacy assistants, physiotherapists, and radiologists to support its operations (19). These facilities have played a critical role in alleviating the burden on medical infrastructure during the surge of the pandemic by offering focused and essential care to COVID-19 patients.

Badahdah et al. (20) reported findings from Oman indicating that HCWs who were in direct contact with COVID-19 patients experienced a higher level of psychological distress and disturbed sleep, compared to their counterparts who were not in direct contact with COVID-19 patients (21). In addition, socio-demographic factors such as gender, age, marital status, socio-economic background, and social support were found to be significant predictors of the psychological distress experienced by frontline HCWs in Oman (7). More specifically, being female, financially unstable, and receiving treatment for mental illness were identified as independent predictors of psychological distress. While many studies have highlighted predictors of psychological distress and the negative impact of the pandemic on HCWs mental wellbeing, others have shown that protective factors, such as resilience, self-efficacy, and healthy coping skills, can improve mental health outcomes (22–24). In fact, a number of studies have reported that HCWs possessed greater resilience despite the adversities associated with the pandemic, including lack of adequate social connection, shift work, lengthy working hours, inadequate hospital supplies, and unpredictability (25). Factors identified as coping mechanisms by HCWs that enhanced their ability to rebound included social support (24, 26), religious activity (27, 28), distraction techniques (27, 29), and adherence to infection control guidelines (27, 30).

Numerous studies from the Arab Peninsula evidenced the negative impact of COVID-19 on HCW’s mental health, however, there is a paucity of data emerging from HCW that operated solely in COVID-19 field Hospitals. As such, the purpose of this study is to address this gap by investigating the mental health status, as indicated by psychological variables and quality of sleep, of HCWs at a COVID-19 field hospital in Oman. The findings of this study may have important implications for policy makers and mental health practitioners in developing effective prevention strategies and policies to reduce the risk of mental health problems among medical responders to the COVID-19 pandemic in Oman, as well as to enhance preparedness for future pandemics. Thus, the present study aims to assess the emotional wellbeing of HCWs at an Omani field hospital by screening them against scales that quantify depression, anxiety, and quality of sleep.

## 2 Methodology

### 2.1 Participants

All 280 HCWs that were operating in the COVID-19 Field Hospital in Oman were invited to participate in the study via social media platforms such as Whatsapp and email services in September 2021. HCWs who were either medical (physicians and nurses) or nonmedical personnel (allied health professionals, pharmacists, and technicians), above 18 years of age, and English or Arabic speakers were eligible to participate. Participants in this cross-sectional study comprised of both Omani and non-Omani HCWs who offered their services at the field hospital any time between October 2020 and September 2021. A sample of 121 HCWs from the COVID-19 field hospital at the Sultanate of Oman gave their consent to participate in this study. Upon obtaining informed consent, all participants were required to complete a 15-min online survey which was shared in both Arabic and English via WhatsApp and email services. The survey comprised of sociodemographic information and questionnaires such as the Patient Health Questionnaire (PHQ-9), the Generalized Anxiety Disorder-7 (GAD-7) and the Pittsburg Sleep Quality Index (PSQI). The screening measures were shared with the standardized instructions to recall experiences and respond to the items in the questionnaires as per the original design. Thus instructions were included for the participants to respond to the PHQ-9 and GAD-7 based on their experiences recalled within the previous 2 weeks, and a recall of 4 weeks for the PSQI.

### 2.2 Measures

#### 2.2.1 Sociodemographic information

A form was given to each participant requesting information such as their age, gender, marital status, nationality, number of dependents, years of medical work experience, time of service at the field hospital, and economic status.

#### 2.2.2 Patient health questionnaire-9

The PHQ-9 is a freely available screening measure comprising of 9 items that is used in the tentative diagnosis of depression and its severity. A cut-off score of 10 on the PHQ-9 was identified as offering a sensitivity of 80.6% and specificity of 94% among Omani HCW (31). For the utilization in this study, a cut-off score of 10 determined the presence of depressive symptoms among HCWs in the sample.

#### 2.2.3 Generalized anxiety disorder-7

The GAD-7 is a freely available, self-report questionnaire comprising of 7 items that requires participants to reflect on their experience of anxiety over the last 2 weeks. The validated questionnaire in Arabic recommends a cut-off score of 10 for more precise sensitivity and specificity of screening for symptomology of anxiety among respondents (32).

#### 2.2.4 Pittsburg sleep quality index

The PSQI comprises of 19-items reporting the quality of sleep experienced over the last month by reviewing various aspects of sleep quality and disturbance. Poor sleep is indicated by any score ranging from 5 to 21on the PSQI with higher scores suggesting a poorer quality of sleep. However in order to ensure the internal consistency and construct validity of the Arabic translation of the PSQI, the cut-off score to mark poor quality of sleep in this study was 3 (33).

### 2.3 Procedure

The study followed a standard procedure for data collection. Owing to social and physical restrictions during the COVID-19 pandemic, HCW who were on the popularly used text-based Smartphone application ‘WhatsApp’ were contacted via the app and given written and verbal information about the study. Participants were requested to complete an online survey using a link on ‘Google Forms’. The form comprised of the information sheet which included information relevant to the study stating the purpose of the study and what their participation will involve. The form also included the following questionnaires: the sociodemographic form, PHQ-9, GAD-7, and PSQI. A debriefing form that restated the study’s purpose and thanked participants for their participation, along with contact details of the researchers was also included in the form. Participants were able to complete the survey at their own pace and at a time that was convenient for them. Once participants completed the survey, they submitted their responses by clicking the “Submit” icon at the bottom of the screen. The responses were forwarded to a secure email that was set up specifically for the purpose of storing the online responses privately. The data was collected and summarized in SPSS V.26 from 121 participants who met the inclusion criteria for the purpose of this study. All completed response sheets were anonymized to maintain confidentiality and compiled into a private dataset that was stored on a password-protected computer. This procedure ensured that the data was kept confidential and secure.

### 2.4 Statistical analysis

The frequency and percentages (%) were calculated for each categorical variable. Continuous variables were described using mean ± standard deviation. The Chi square test was used to test the association between demographic variables and anxiety, depression, and quality of sleep. Binary logistic regression tests were used to test the effect of quality of sleep on anxiety and depression. In all tests, significant levels of 0.05 (*p* = 0.05) were used. For the analysis, we used the Statistical Package for Social Science (SPSS v.26).

### 2.5 Ethics statement

The present research received ethical approval from the Health Studies and Research Approval Committee (HSRAC), Ministry of Health Oman, proposal ID: MoH/CSR/22/26365. All participants provided informed written consent prior to participating in the study. They were required to read through an information sheet summarizing the aims of the study and how their data will be stored. They were informed of their right to withdraw from the study at any point and that they have the choice to delete their responses even after completing the survey. They were also made aware that all their information would remain anonymous and may not be tracked back to them in any way. Finally, participants were informed that there were no expected risks or harm associated with participating in the study. However, to mitigate any risk to their mental well-being as a result of participating in the study, the researchers included mental health resources and contact emails of the authors in the information sheet and debrief form.

## 3 Results

A total of 121 healthcare workers (HCWs) from the COVID-19 Field hospital in Oman participated in this study, offering insight into the sociodemographic profiles and various characteristics within a field hospital setting. The distribution of gender among participants skewed toward females, who comprised 63.6% (*n* = 77) of the sample, compared to 36.4% males (*n* = 44). This gender disparity highlights the prominent role of female HCWs in the healthcare setting under study and may reflect broader trends within the healthcare profession or specific recruitment patterns of this study.

Age distribution among participants indicates a significant concentration in the 30–39 years age bracket, with 79.4% (*n* = 96) of the sample falling within this range. This suggests that the workforce is relatively young, which could have implications for the energy levels, technological adaptability, and possibly the stress resilience of the workforce. The relatively smaller proportions of younger (13.2%, *n* = 16) and older (7.4%, *n* = 9) workers suggest less diversity in age, which might influence the dynamics of teamwork and peer support within the hospital.

Regarding marital status, a vast majority of participants were married (82.7%, *n* = 100), with single individuals accounting for 12.4% (*n* = 15), and very few divorced (0.8%, *n* = 1) or engaged (4.1%, *n* = 5). The high prevalence of married individuals could indicate a need for work-life balance considerations and support structures for family obligations among the workforce.

Nationality data reveal that 90.9% (*n* = 110) of the participants were non-Omani, highlighting a workforce predominantly composed of expatriates. This has significant implications for cultural diversity and possibly the adaptation challenges and social support systems available to the HCWs in the field hospital.

The number of dependents shows a roughly even split between those with fewer than 3 dependents (47.0%, *n* = 47) and those with 3 to 5 dependents (48.0%, *n* = 48), with a small fraction having more than 5 dependents (5.0%, *n* = 5). This distribution suggests a significant portion of the workforce faces considerable family responsibilities outside of their professional roles.

Work experience, measured as an average of 7.22 years with a standard deviation of 4.98, indicates a moderately experienced workforce. The range in years of experience suggests diversity in the level of expertise and possibly differences in approaches to patient care and adaptability to the field hospital’s demanding environment.

The time spent in the field hospital shows that a large majority of participants (73.6%, *n* = 90) have been working for 6–9 months, with a notable group (24.0%, *n* = 29) exceeding 10 months. This indicates that most of the workforce has had a substantial duration of direct exposure to the specific challenges of the field hospital setting.

Economically, 65.2% (*n* = 79) of participants reported unstable financial status, with only 32.3% (*n* = 39) feeling comfortable financially. This suggests that economic concerns are prevalent among the workforce, which could have implications for their mental health and overall well-being (Table 1).

**Table 1:** Sociodemographic data and other characteristics of the sample.

Category	N (%)
Gender	
Male	44 (36.4%)
Female	77 (63.6%)
Age	
18–29	16 (13.2%)
30–39	96 (79.4%)
40–50	9 (7.4%)
Marital Status	
Single	15 (12.4%)
Married	100 (82.7%)
Divorced	1 (0.8%)
Engaged	5 (4.1%)
Nationality	
Omani	11 (9.1%)
Non- Omani	110 (90.9%)
Number of Dependents	
< 3 dependents	47 (47.0%)
3–5 dependents	48 (48.0%)
> 5 dependents	5 (5.0%)
Years of work experience	
(Mean ± SD)	7.22 ± 4.98
Time Spent in Field Hospital	
< 2 Months	0 (0.0%)
2–5 Months	2 (1.7%)
6–9 Months	90 (73.6%)
>10 Months	29 (24.0%)
Economic Status	
Comfortable Financially	39 (32.3%)
Financial Status Unstable	79 (65.2%)
Very Comfortable Financially	3 (2.5%)
Depression	
Yes	9 (7.4%)
No	112 (92.6%)
Anxiety	
Yes	9 (7.4%)
No	112 (92.6%)
Quality of sleep Problem	
Yes	49 (40.5%)
No	72 (59.5%)

*N* = 121.

The prevalence of depression and anxiety was reported at 7.4% for both conditions (*n* = 9 for each), indicating that these mental health challenges, while not dominant, are present among the HCWs. The quality of sleep problem was more pronounced, with 40.5% (*n* = 49) of the participants reporting issues, highlighting a significant area of concern for worker health and performance.

In summary, the sociodemographic and characteristic profile of the HCWs in this study provides critical insights into the workforce’s composition and potential needs. The predominance of a young, predominantly female, and married workforce, with a significant portion experiencing financial instability and sleep quality issues, points to several areas for policy intervention, including mental health support, financial counseling, and initiatives to improve work-life balance and well-being.

Table 2 examines the prevalence of GAD among healthcare workers (HCWs) in relation to demographic and work-related variables. Notably, a significant difference in anxiety levels is observed based on marital status and nationality, with engaged HCWs experiencing higher anxiety rates (100%) and Omani nationals showing a markedly higher prevalence of anxiety (45.5%) compared to their non-Omani counterparts (3.6%). These findings suggest that social and cultural factors may significantly influence mental health outcomes among HCWs. Additionally, economic status and time spent in the field hospital were significantly related to anxiety levels, indicating that financial instability and prolonged exposure to the field hospital environment may exacerbate anxiety among HCWs. The relatively high anxiety rates among those with a “comfortable” financial status (15.8%) compared to the “financially unstable” group (2.6%) underscore the complex relationship between perceived economic well-being and mental health.

**Table 2:** Generalized anxiety disorder based on several variables.

Category	HCW without anxiety N (%)	HCW with anxiety N (%)	*p* value
Gender			
Male	43 (97.7%)	1 (2.3%)	<0.102
Female	69 (89.6%)	8 (10.4%)	
Age			
18–29	13 (81.3%)	3 (18.8%)	
30–39	91 (94.8%)	5 (5.2%)	<0.146
40–50	8 (88.9%)	1 (11.1%)	
Marital Status			
Single	81 (95.3%)	4 (4.7%)	
Married	26 (86.7%)	4 (13.3%)	<0.002*
Divorced	5 (100%)	0 (0%)	
Engaged	0 (0%)	1 (100%)	
Nationality			
Omani	6 (54.5%)	5 (45.5%)	<0.000*
Non- Omani	106 (96.4%)	4 (3.6%)	
Number of Dependents			
< 3 dependents	42 (89.4%)	5 (10.6%)	
3–5 dependents	45 (93.8%)	3 (6.3%)	<0.401
> 5 dependents	3 (75%)	1 (25%)	
Years of work experience			
(Mean ± SD)	7.17 ± 5.01	7.79 ± 4.91	<0.722
Time Spent in Field Hospital			
< 2 Months	1 (50%)	1 (50%)	
2–5 Months	86 (95.6%)	4 (4.4%)	<0.017*
6–9 Months	25 (86.2%)	4 (13.8%)	
>10 Months	1 (50%)	1 (50%)	
Economic Status			
Comfortable Financially	32 (84.2%)	6 (15.8%)	
Financial Status Unstable	76 (97.4%)	2 (2.6%)	<0.009*
Very Comfortable Financially	2 (66.7%)	1 (33.3%)	
Depression			
No	108 (96.4%)	4 (3.6%)	<0.000*
Yes	4 (44.4%)	5 (55.6%)	
Quality of sleep Problem			
No	70 (98.6%)	1 (1.4%)	<0.002*
Yes	40 (83.3%)	8 (16.7%)	

*N* = 121. *p* = 0.05.

*Significant level at 0.05.

Table 3 focuses on the association between depressive symptoms and various sociodemographic factors among HCWs. The analysis reveals a statistically significant relationship between age and depression, with younger HCWs aged 18–29 and those aged 40–50 showing higher rates of depression compared to the 30–39 age group. This suggests that the youngest and oldest segments of the workforce may be more susceptible to depression, possibly due to different stressors and challenges faced at these career stages. Nationality again plays a critical role, with Omani HCWs showing a higher prevalence of depressive symptoms (45.5%) than non-Omanis (3.6%), highlighting the potential impact of cultural and societal factors on mental health. The relationship between time spent in the field hospital and depression, particularly among those with shorter and longer durations, suggests that the initial adjustment period and long-term exposure to stressful work environments may contribute to depressive symptoms.

**Table 3:** Relationship between depressive symptoms and sociodemographic variables.

Category	HCW without depression N (%)	HCW with depression N (%)	*p* value
Gender			
Male	43 (97.7%)	1 (2.3%)	<0.102
Female	69 (89.6%)	8 (10.4%)	
Age			
18–29	13 (81.3%)	3 (18.8%)	
30–39	92 (95.8%)	4 (4.2%)	<0.026*
40–50	7 (77.8%)	2 (22.2%)	
Marital Status			
Single	81 (95.3%)	4 (4.7%)	
Married	25 (83.3%)	5 (16.7%)	<0.164
Divorced	5 (100%)	0 (0%)	
Engaged	1 (100%)	0 (0%)	
Nationality			
Omani	6 (54.5%)	5 (45.5%)	<0.000*
Non- Omani	106 (96.4%)	4 (3.6%)	
Number of Dependents			
< 3 dependents	43 (91.5%)	4 (8.5%)	
3–5 dependents	45 (93.8%)	3 (6.3%)	<0.778
> 5 dependents	4 (100%)	0 (0%)	
Years of work experience			
(Mean ± SD)	7.06 ± 4.77	9.06 ± 7.01	<0.251
Time Spent in Field Hospital			
< 2 Months	0 (0.0%)	0 (0.0%)	
2–5 Months	1 (50%)	1 (50%)	<0.048*
6–9 Months	85 (94.4%)	5 (5.6%)	
>10 Months	26 (89.6%)	3 (10.4%)	
Economic Status			
Comfortable Financially	33 (86.8%)	5 (13.2%)	
Financial Status Unstable	75 (96.2%)	3 (3.8%)	<0.048*
Very Comfortable Financially	2 (66.7%)	1 (33.3%)	
Anxiety			
No	108 (96.4%)	4 (3.6%)	<0.000*
Yes	4 (44.4%)	5 (55.6%)	
Quality of sleep Problem			
No	70 (98.6%)	1 (1.4%)	<0.002*
Yes	40 (83.3%)	8 (16.7%)	

*N* = 121, *p* = 0.05.

*Significant level at 0.05.

Table 4 examines the impact of sociodemographic factors on the quality of sleep among HCWs. A notable finding is the significant difference in sleep quality based on nationality, with Omani HCWs experiencing more sleep difficulties (81.8%) compared to non-Omanis (36.1%). This might reflect cultural, environmental, or work-related differences affecting sleep. The table also indicates that age and gender may influence sleep quality, with older HCWs and females reporting more sleep problems. The correlation between generalized anxiety disorder and sleep difficulties is strongly evident, with those suffering from anxiety much more likely to experience sleep problems (88.9% with sleep difficulties), suggesting a close interrelation between anxiety and sleep quality.

**Table 4:** Relationship between quality of sleep and sociodemographic variables.

Grouping	HCW without sleep difficulties N (%)	HCW with sleep difficulties N (%)	*p* value
Gender			
Male	31 (70.5%)	13 (29.5%)	<0.064
Female	41 (53.3%)	36(46.7%)	
Age			
18–29	11 (68.2%)	5 (33.3%)	
30–39	59 (61.5%)	37 (38.5%)	<0.052
40–50	2 (25%)	6 (75%)	
Marital Status			
Single	52 (61.9%)	32 (38.1%)	
Married	14 (50.0%)	15 (50.0%)	<0.107
Divorced	5 (100%)	0 (0%)	
Engaged	0 (0%)	1 (100%)	
Nationality			
Omani	2 (18.2%)	9 (81.8%)	<0.000*
Non- Omani	70 (63.9%)	40 (36.1%)	
Number of Dependents			
< 3 dependents	30 (63.8%)	17 (36.2%)	
3–5 dependents	26 (55.3%)	21 (44.7%)	<0.286
> 5 dependents	1 (25%)	3 (75%)	
Years of work experience			
(Mean ± SD)	6.79 ± 4.61	7.65 ± 5.33	<0.266
Time Spent in Field Hospital			
< 2 Months	0 (0.0%)	0 (0.0%)	
2–5 Months	1 (50%)	1 (50%)	<0.954
6–9 Months	54 (60.2%)	36 (39.8%)	
>10 Months	17 (58.6%)	12 (41.4%)	
Economic Status			
Comfortable Financially	19 (50.0%)	19 (50.0%)	
Financial Status Unstable	52 (66.7%)	26 (33.7%)	<0.141
Very Comfortable Financially	1 (33.3%)	2 (66.7%)	
Generalized Anxiety Disorder			
No	70 (63.6%)	40 (36.4%)	<0.002*
Yes	1 (11.1%)	8 (88.9%)	
Quality of sleep Problem			
No	70 (63.6%)	40 (36.4%)	<0.002*
Yes	1 (11.1%)	8 (88.9%)	

*N* = 121, *p* = 0.05.

*Significant level at 0.05.

The analysis across Tables 2–4 illuminates the intricate web of factors influencing anxiety, depression, and sleep quality among healthcare workers. Marital status, nationality, economic status, and the duration of time spent in a high-stress field hospital environment emerge as significant determinants of mental health and well-being. The higher prevalence of anxiety and depression among certain demographic groups, particularly Omani HCWs, points to the need for targeted mental health interventions. Moreover, the clear association between anxiety and poor sleep quality underscores the importance of addressing mental health issues as part of holistic approaches to improving the overall health and productivity of the healthcare workforce.

The core of our analysis centers on a binary dependent variable—quality of sleep (problematic vs. non-problematic), our study delves into the impacts of sleep quality on psychological distress, namely anxiety, depression and nationality. This specificity, combined with our distribution, situates our study within a framework where traditional Event Per Variable (EPV) considerations are met for our one predictor logistic regression model. According to the widely acknowledged EPV rule, a minimum of 10 events per predictor variable is recommended to ensure stable parameter estimates. Our dataset exceeds this threshold, with more than 24 events for each category per predictor, ostensibly aligning with the basic statistical requirements for logistic regression analysis. This study by Peduzzi et al. (34) is pivotal because it investigates the effect of the number of events per variable (EPV) on the reliability of logistic regression coefficients, concluding that a minimum of 10 events per predictor is needed for reliable estimates. This rule of thumb has been widely adopted in statistical analyses to guide sample size and predictor selection in logistic regression models, thereby underpinning the methodological foundation of our study and reinforcing the adequacy of our sample distribution for the intended analysis (Table 5).

**Table 5:** Logistic regression results for predictors of psychological distress.

	B	S.E.	Wald	df	Sig.	Exp(B)	Cox & Snell R Square
GAD	2.110	0.541	15.207	1	0.000	8.246	0.238
Nationality	1.795	1.172	2.345	1	0.126	6.019	
Constant	−0.866	0.234	13.744	1	0.000	0.421	

A logistic regression analysis was conducted to explore factors influencing PSQI (likely indicating presence/absence of a condition). The results revealed a strong positive association between General Depression and Anxiety Disorder (GAD) and PSQI. For every one-unit increase in GAD score, the odds of having a positive PSQI score increased by a factor of 8.246 (*p*-value = 0.000). This statistically significant finding suggests that individuals with higher GAD scores are significantly more likely to have a positive PSQI score.

The effect of nationality on PSQI, however, was not statistically significant (*p*-value = 0.126). While the Exp (B) value of 6.019 suggests a possible positive association, more data or a stronger effect size may be necessary for confirmation. The constant term (−0.866) represents the model’s intercept and does not hold direct meaning in the context of PSQI.

The Cox & Snell R-squared value of 0.238 indicates that the logistic regression model explains approximately 24% of the variance in predicting positive PSQI scores. While this does not necessarily imply a “good” fit (as the benchmark for goodness-of-fit can vary by field), it suggests the model provides a moderate level of explanation for PSQI based on GAD and nationality. It is important to consider additional goodness-of-fit measures and potentially explore including more explanatory variables to improve the model’s predictive power.

In conclusion, this study highlights a strong positive association between GAD and PSQI, suggesting that individuals with GAD are more likely to have a positive PSQI score. The influence of nationality remains inconclusive and warrants further investigation.

## 4 Discussion

### 4.1 Main findings

This study aimed to evaluate the emotional status of medical front line workers that were assigned to the COVID-19 field hospital in the Sultanate of Oman with the use of surveys. The ‘emotional status’ of HCWs was evaluated by quantifying the level of subjective depression, anxiety and the general quality of sleep experienced by HCWs. Participants in the study were HCW and were inclusive of “medical” (physicians and nurses) and “nonmedical” personnel (allied health professionals, pharmacists, and technicians).

Similar to studies from Saudi Arabia, Iran and Turkey, the present study found that the majority of the sample did not present with features that indicated depression, anxiety, or poor quality of sleep as a result of their work in the field hospital (35, 36). This finding aligns with existing literature globally and from the Arab region that have found that HCWs possess resilience and may not succumb to overwhelming distress during times of crisis (37). While it has been largely reported that the prevalence of anxiety disorders and depression are higher among HCWs during the COVID-19 pandemic, it is possible that scores in the present study might be attributed to mental preparedness as a result of emerging literature globally at the time and the infection control measures that were in place at the field hospital in Oman (38). Studies have suggested multiple protective factors that impact one’s perception of the work environment, thereby enhancing resilience (39). Some of these factors include teamwork, acts of appreciation, dependability, dedication to the job, emotional support, and self-efficacy (40, 41).

In addition to accounting for potential confounding variables such as marital status and socio-economic status, the present study also identified a significant association between the duration of employment in the field hospital and the level of anxiety experienced by HCWs. Specifically, HCWs who had been employed at the field hospital for less than 5 months exhibited higher levels of anxiety compared to those who had been employed for longer durations. These findings are consistent with previous research suggesting that prolonged work exposure, lengthy working hours, sense of urgency, and increased workload can contribute to symptoms of burnout among HCWs (42–44). However, more recent data has emerged highlighting the resilience of HCWs and their ability to adapt to challenging situations (25). It is possible that the present study’s findings reflect this resilience, with HCWs becoming more emotionally resilient over time as they adjust to their work environment and develop coping strategies. Further research is needed to better understand the protective factors that contribute to HCWs’ resilience and ability to cope with challenging work environments, as this knowledge can inform decision-making and interventions aimed at protecting the well-being of HCWs during global health emergencies such as the COVID-19 pandemic.

In addition, prolonged stress or anxiety response in the data suggested a higher risk of poor quality of sleep. This has been reflected in findings from studies emerging from Austria (45), Turkey (46), and Italy (47) which all demonstrated that elevated and prolonged levels of stress and anxiety are associated with poorer sleep quality. Interestingly, in contrast to these studies, the present study reported minimal impact on HCWs quality of sleep as a result of ‘pandemic fatigue’. A study from the Philippines (48) found similar results among nurses that worked directly with COVID-19 patients. The implementation of safety protocols, increased vaccination regulations, and a positive outlook toward clinical resources are likely to contribute to lower levels of pandemic fatigue and more adequate sleep quality among medical frontliners.

Despite the increase in stressors and psychological vulnerability during the peak of the pandemic in 2020 within Oman, the findings of the current study are consistent with studies of resilience among medical HCW globally as the situation developed. While the events may be considered ‘traumatic’, trauma may not always yield a negative outcome, and this is subjective to the protective factors and personal resources that one is given access to at the time. Authors from a study in Madrid identified that resilience was vital to the psychological safety of HCW and enables them to cope with the increasing pressures of the role (49). The full extent of the psychological and physical toll that this pandemic may have had on the HCWs is yet to be fully understood. The findings of the present study have shed light on the enhanced emotional resilience of HCWs that were on the frontlines of the COVID-19 pandemic in the Sultanate of Oman. Further research into the protective factors and psychological status of the HCW would be helpful as it can inform governmental decision-making and further protect the welfare of HCW, whose role is vital during global health emergencies such as the COVID-19 pandemic.

### 4.2 Limitations and strengths

The findings of the study must be interpreted with caution as there are several limitations to consider, which highlight the need for further investigation. The data was gathered using self-reported questionnaires which are not the most reliable source of data collection; hence, the responses are subject to biases, and they were not verified against medical records. Furthermore, the responses may be subject to the interpretation of distress based on cultural and ethnic backgrounds. In the present study, the sample comprised of HCWs who were both medical and non-medical personnel which may differ in terms of exposure to the virus and being in first contact with COVID-19 patients. Medical personnel have different responsibilities to non-medical personnel and may be more overwhelmed by their duties compared to non-medical personnel. The sample was not analyzed separately which may have influenced the results. A larger sample may have yielded more accurate and reliable results. Furthermore, additional background variables such as prior history of mental illness could have further informed the findings in this study.

However, a significant strength of this study is that it is one of the few studies conducted on HCWs working in field hospitals, which is a unique and challenging environment. Additionally, the study contributes to the growing body of literature on the impact of the COVID-19 pandemic on HCWs’ mental health and well-being. Moreover, the study’s findings highlight the enhanced emotional resilience of HCWs in Oman, which is an important factor in managing the increasing pressures of their role during global health emergencies. Lastly, the study provides insights into the protective factors that can help HCWs cope with the psychological and physical toll of their work.

To address the limitations of the present study, future research could employ more objective measures of stress and anxiety, such as physiological indicators or clinician diagnoses, to validate the self-reported data. In addition, future studies could address the limitations of this study by using more objective measures of data collection such as medical records or biomarkers to confirm the self-reported results and also utilize the qualitative methodology to identify more robust data. Additionally, analyzing medical and non-medical personnel separately to explore any differences in their experiences. Future studies could also explore the long-term effects of the pandemic on HCWs’ mental health and well-being, as well as the role of organizational support and resources in promoting resilience among HCWs.

## 5 Conclusion

To our knowledge, this study is the first to evaluate the emotional status of HCWs from a field hospital in the Sultanate of Oman. The findings of this study have presented the opportunity for further follow-up studies to evaluate any long-term psychological impact of the pandemic. Furthermore, this study underscores the global resilience observed among healthcare workers, prompting a call for further examination into the role of socio-cultural phenomena. Exploring the impact of factors such as strong family ties, religious views, effective leadership, and community cohesion on the resilience of healthcare workers could offer valuable insights. Evaluating how these elements contribute to their ability to cope with stress and challenging work environments can provide valuable insights. Such insights can guide policymakers in reinforcing these protective factors to better support healthcare workers in any future incidents of public health crises.

## Data availability statement

The raw data supporting the conclusions of this article will be made available by the authors, without undue reservation.

## Ethics statement

The studies involving humans were approved by the Health Studies and Research Approval Committee (HSRAC), Ministry of Health Oman, proposal ID: MoH/CSR/22/26365. The studies were conducted in accordance with the local legislation and institutional requirements. The participants provided their written informed consent to participate in this study.

## Author contributions

RM: Writing – review & editing, Writing – original draft, Conceptualization, Data curation, Investigation, Methodology. HA-S: Writing – original draft, Writing – review & editing, Conceptualization, Methodology. NA-K: Writing – original draft, Writing – review & editing, Conceptualization, Investigation. SR: Writing – original draft, Writing – review & editing, Project administration, Supervision. RB: Writing – review & editing, Writing – original draft, Data curation, Formal analysis, Methodology. NA-L: Writing – review & editing, Methodology, Supervision. MR: Writing – review & editing, Project administration. KA-R: Writing – review & editing. AM: Writing – original draft, Writing – review & editing, Formal analysis, Methodology, Software.
